# Stomach Metastasis in a Patient with Prostate Cancer 4 Years after the Initial Diagnosis: A Case Report and a Literature Review

**DOI:** 10.1155/2012/292140

**Published:** 2012-12-01

**Authors:** Ahmet Bilici, Mustafa Dikilitas, Ozlem Ton Eryilmaz, Bekir Selim Bagli, Fatih Selcukbiricik

**Affiliations:** ^1^Department of Medical Oncology, Sisli Etfal Education and Research Hospital, Sisli, 34210 Istanbul, Turkey; ^2^Department of Pathology, Sisli Etfal Education and Research Hospital, Sisli, 34210 Istanbul, Turkey

## Abstract

Prostate cancer commonly metastasizes bones and lymph nodes, but it very rarely spreads to the gastrointestinal tract. However, only five cases of prostate cancer metastatic to the stomach have been previously reported in the literature. We report a case of a 69-year-old man with metastatic prostate cancer who presented with upper gastrointestinal bleeding (UGB) 4 years after the diagnosis. Esophagogastroscopy revealed multiple ulcerations in the gastric body and histopathological examination confirmed gastric metastasis that originated from prostate cancer. Chemotherapy could not be given because of patient's refusal. He was treated with LHRH agonist. We suggest that for a man with prostate cancer diagnosed with UGB, stomach metastasis should be considered in the differential diagnosis of UGB.

## 1. Introduction

Although the bones and lymph nodes are the most common sites for distant metastasis in prostate cancer, it rarely metastasizes the lungs and liver [1]. The involvement of gastrointestinal tract is also very rare and only five cases of prostate cancer metastatic to the stomach have been previously reported in the literature [2–5]. Herein, we described the unusual case of prostate cancer metastasized to the stomach as the manifestation of recurrence and reviewed the literature. 

## 2. Case Report

**Figure 1:**
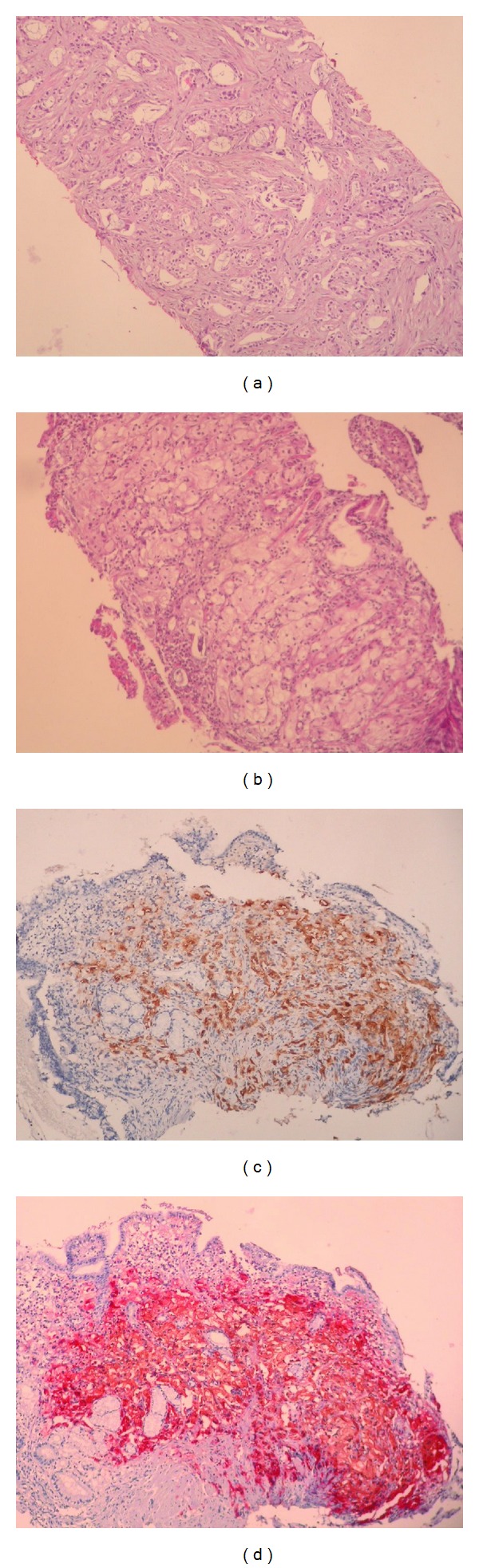
(a) Prostatic needle core biopsies show Gleason's grade 4 prostatic adenocarcinoma. (hematoxylin-eosin stain, ×200). (b) The gastric biopsy reveals infiltration of the neoplastic cells with round nuclei and abundant eosinophilic to amphophilic cytoplasm. (hematoxylin-eosin stain, ×200). (c) The neoplastic cells strongly immunoreactive for PSA (Immunohistochemical stain for PSA, ×200). (d) The neoplastic cells strongly immunoreactive for PSAP (immunohistochemical stain for PSAP, ×200).

 A 69-year-old man was presented with our hospital in October 2007, with four-month history of anorexia, painless gross hematuria, and back pain. He had a history of paranoid schizophrenia for fifty years and he was taking olanzapine 10 mg/day p.o. His family history was non-contributory. Physical examination was normal except for tenderness at the thoracic vertebrates. The digital rectal examination revealed a firm, slightly enlarged prostate. Initial laboratory results were as follows: white blood cell (WBC) 4700/mm^3^, platelets 202000/mm^3^, hematocrit 34.2%, MCV 80.4 fL, and PSA 89 ng/mL. Other laboratory values were within normal limits. Urologic ultrasonography showed grade 1-2 hydronephrosis on the left kidney and slightly enlarged prostate gland. A bone scan revealed multiple bone metastasizes in the thoracic vertebrates (T_6_, T_7_, T_8_ and T_11_) and the left iliac bone and femur. Thorax and abdominopelvic CT scans were normal for other metastases. The prostate biopsy was performed and histopathology confirmed an adenocarcinoma of prostate, with a Gleason score of 7 (3 + 4) (Figure 1(a)). Initially because of painful bone metastases, radiotherapy was given. Thereafter, he was treated with antiandrogen therapy with goserelin acetate 10.8 mg implant, every 12 weeks, and zoledronic acid 4 mg i.v., every 28 days. After initiating hormonal ablation, the PSA level decreased and then returned to normal limits within three months.

 While the patient was remained in the remission during a followup of 4-years, he was referred to our clinic with hematemesis and melena which started one day ago, in July 2011. Physical examination revealed normal systems, findings except for mild hypotension (95/60 mm/Hg) and tachycardia (104/min). In addition the rectal examination showed melena. Initial laboratory results were as follows: WBC 6800/mm^3^, platelets 202000/mm^3^, hematocrit 20.7%, MCV 89.5 fL, urea 87 mg/dL, creatinine 2.1 mg/dL, and PSA 244.8 ng/mL. A fecal occult blood test was positive. Other laboratory values were within normal limits. Initially he was treated with erythrocyte transfusion and i.v. H_2_-receptor blocker. Esophagogastroscopy revealed multiple ulcerations in the gastric body. Histopathological examination of endoscopic biopsies showed an infiltration of the neoplastic cells with round nuclei and abundant eosinophilic to amphophilic cytoplasm (Figure 1(b)). Immunohistochemically the neoplastic cells were positive for PSA (prostate-specific antigen) and PSAP (prostatic-specific acid phosphatase) and negative for cytokeratin 7 and cytokeratin 20 (Figures 1(c) and 1(d)). The pathologic findings supported a diagnosis of gastric metastasis of metastatic prostatic adenocarcinoma.

Chest and abdominopelvic CT scans were negative for distant metastasis except for bone metastasis. After that bicalutamide 50 mg/day p.o. was added to goserelin acetate. The PSA level was decreased to 122 ng/mL during three months, but it was increased to 837.4 ng/mL and total testosterone level measured was <20 ng/dL after six months. In the light of these findings, the patient was thought as hormone resistant and bicalutamide was discontinued. Chemotherapy with docetaxel was planned, but it could not be given because of patient's refusal. Therefore, the treatment with LHRH agonist and zoledronic acid was maintained. He had no specific symptom and was remained in stable disease stage, during a followup of 7 months after the diagnosis of gastric metastasis. 

## 3. Discussion

 Although prostate cancer can metastasize to almost any site in the body, lymph nodes and bones remain the most common sites of metastasis [1]. However, its metastasis to the gastrointestinal tract is very unusual and small bowel [6] and esophageal metastasis [7, 8] secondary to prostate cancer have been also rarely documented. In addition, prostate cancer metastasis to stomach has also been rarely reported in the literature [2–5]. Two postmortem studies showed that the incidence of gastric metastasis originated from metastatic prostate cancer was 1% to 4% [9, 10]. 

The most commonly reported primary malignancies in the literature to result in gastric metastasis are lung, pancreas, esophagus, liver, breast, kidney cancer, and colon carcinoma [9, 11–13], but only five cases of gastric metastasis from prostate cancer have been previously reported [2–5]. In two of the patients, gastric metastasis was an initial finding at the diagnosis of prostate cancer [3, 4]. On the other hand, the diagnosis of gastric metastasis has been made months or years after the diagnosis of prostate cancer in the remaining three patients [2, 4, 5] and our case. In other words, previous reports and our case indicated that gastric metastasis was commonly seen as a finding of relapse in prostate cancer. Therefore, our patient was compatible with the literature. 

 Nausea, vomiting, and abdominal or epigastric discomfort were commonly presenting symptoms in the majority of reported cases with gastric metastasis [2–5]. However, hematemesis in case 4 [4] and upper gastrointestinal bleeding with hematemesis and melena in our case were initial symptoms for gastric metastasis. Green showed in her study that most common initial symptoms or findings for gastric metastasis from solid tumors were diffuse abdominal pain, nausea and vomiting, anorexia, guaiac-positive stool, and gastrointestinal bleeding, respectively [9]. The features of cases with gastric metastasis of prostate cancer are summarized in Table 1. 

 The median time to gastric metastasis was 33 months (range 15–96 months) for case 1, 4, 5, and this case. In our patient and previous reported cases, PSA levels at the diagnosis of gastric metastasis were elevated [2–5], but it was not applicable in case 5 [5]. The majority of previous cases and our cases had hormone-refractory prostate cancer with other distant metastases and TAB was mostly used treatment modalities except for case 5 [5]. Our case was also treated with TAB because he refused chemotherapy. Although chemotherapy could not be given, he remained in stable disease stage.

The mechanism of metastasis of gastrointestinal tract secondary to prostate cancer is controversial. Hematogenous, lymphatic, and direct extension of primary tumor may lead to metastasis [14]. Moreover, predominant tumor for metastases such as lung or liver had rich capillary vessels and have a constant blood flow, but metastasis to gastrointestinal tract may occur via the lymphatic route because the prostate had rich lymphatic drainage [14]. 

This paper constitutes the unusual case of prostate cancer metastasized to stomach in the literature. In patients with prostate cancer who presented with upper gastrointestinal bleeding or severe nausea and vomiting, gastric metastasis of prostate cancer should be considered in the differential diagnosis of gastrointestinal bleeding as the other causes. 

## Figures and Tables

**Table 1:** The features of cases with gastric metastasis of prostate cancer.

Case	Age, y	Stage at the diagnosis	Gleason's score	Initial presentation of gastric metastasis	PSA level at the gastric metastasis (ng/mL)	Time to gastric metastasis (months)	Treatment	Reference
1	88	NA	2 + 5 (7)	Postprandial vomiting and epigastric discomfort	800	96	NA	[2]
2	67	Metastatic	NA	Severe nausea and vomiting	171	Initial finding	TAB	[3]
3	89	Metastatic	NA	Nausea, vomiting, and decreased appetite	1565	Initial finding	TAB	[4]
4	57	Early stage	5 + 4 (9)	Hematemesis	240	15	TAB	[4]
5	66	Locally advanced	5 + 4 (9)	Nausea, vomiting, and abdominal discomfort	NA	18	Chemotherapy	[5]
This paper	69	Metastatic	3 + 4 (7)	UGB	244.8	48	TAB	—

*PSA: prostate-specific antigen; TAB: total androgen blockade; UGB: upper gastrointestinal bleeding; NA: not applicable.
